# Supplementary material for: Selective CO2 electrolysis to CO using isolated antimony alloyed copper
Source: Nat Commun. 2023 Jan 20;14:340. doi: 10.1038/s41467-023-35960-z (PMC9860050; doi:10.1038/s41467-023-35960-z)
Supplement: Supplementary file 1 — Supplementary Information [file 41467_2023_35960_MOESM1_ESM.pdf]

Supplementary Material for

Selective CO_2 electrolysis to CO using isolated antimony alloyed copper

Jiawei Li†, Hongliang Zeng†, Xue Dong†, Yimin Ding, Sunpei Hu, Runhao Zhang, Yizhou Dai, Peixin Cui, Zhou Xiao, Donghao Zhao, Liujiang Zhou, Tingting Zheng, Jianping Xiao*, Jie Zeng*, Chuan Xia*

*Corresponding authors: xiao@dicp.ac.cn (J.X.); zengj@ustc.edu.cn (J.Z.); chuan.xia@uestc.edu.cn (C.X.)

†These authors contributed equally to this work.

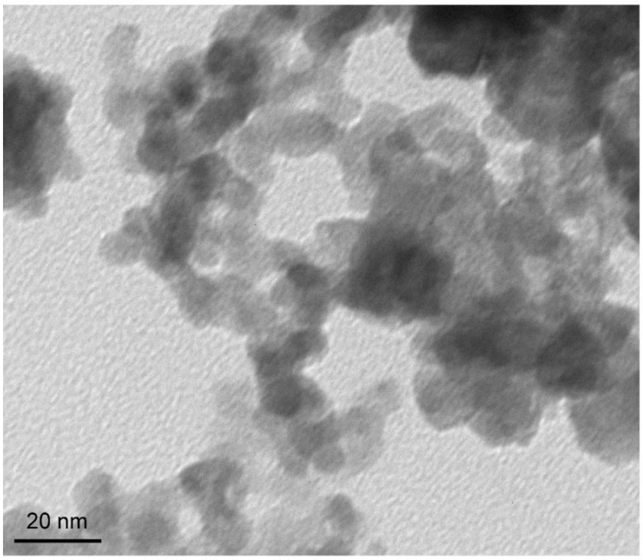

Supplementary Fig. 1 | TEM image of Sb_1Cu-5. The image showed nanoparticles with a homogeneous morphology and sizes ranging from 10 to 20 nm.

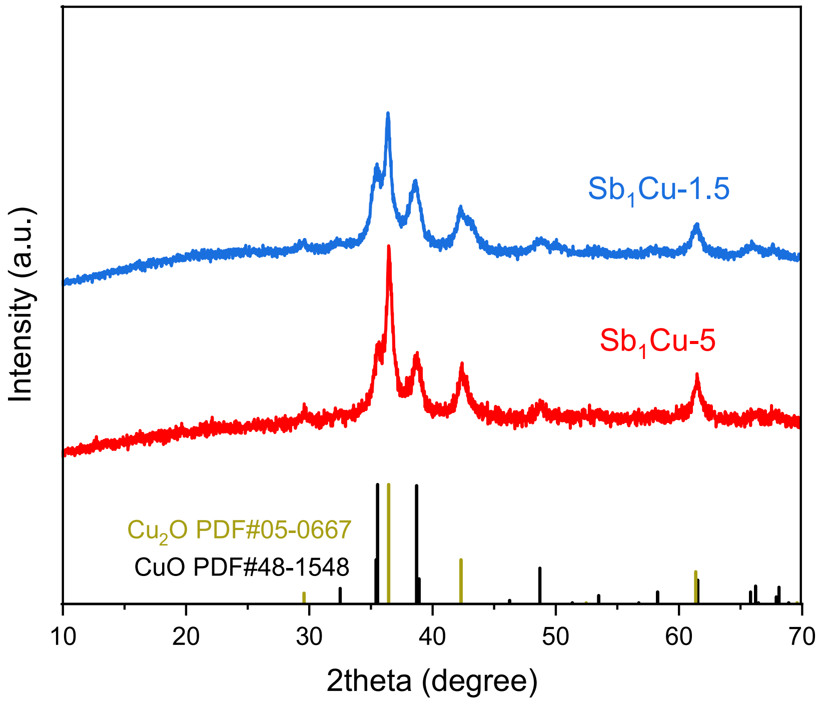

Supplementary Fig. 2 | PXRD patterns of as-prepared Sb_1Cu catalysts. The peaks were consistent with standard Cu_2O and CuO. No Sb or Sb oxides diffraction peaks were found, excluding the formation of Sb nanoparticles. The formation of copper oxides was due to the fact that Cu nanocrystal surface is oxygen susceptible when exposed to air.

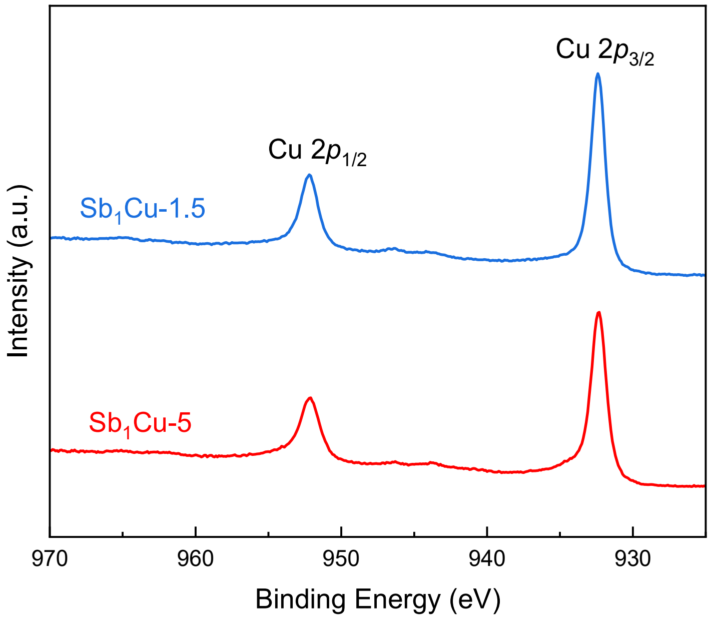

Supplementary Fig. 3 | Cu 2*p* XPS spectra of as-prepared Sb_1Cu catalysts. The peaks at 952.2 eV and 932.3 eV were assigned to Cu 2$p_{1/2}$ and Cu 2$p_{3/2}$, respectively.

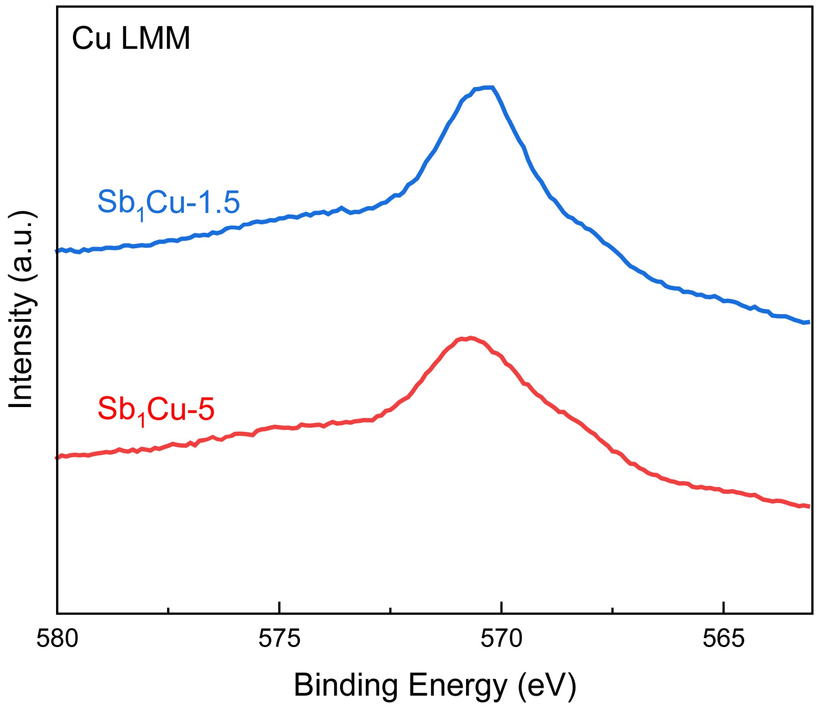

Supplementary Fig. 4 | Cu LMM Auger XPS spectra of as-prepared Sb_1Cu catalysts. The peaks at approximately 570 eV were assigned to Cu^{I}, confirming the spontaneous oxidation of the as-prepared Sb_1Cu catalysts.

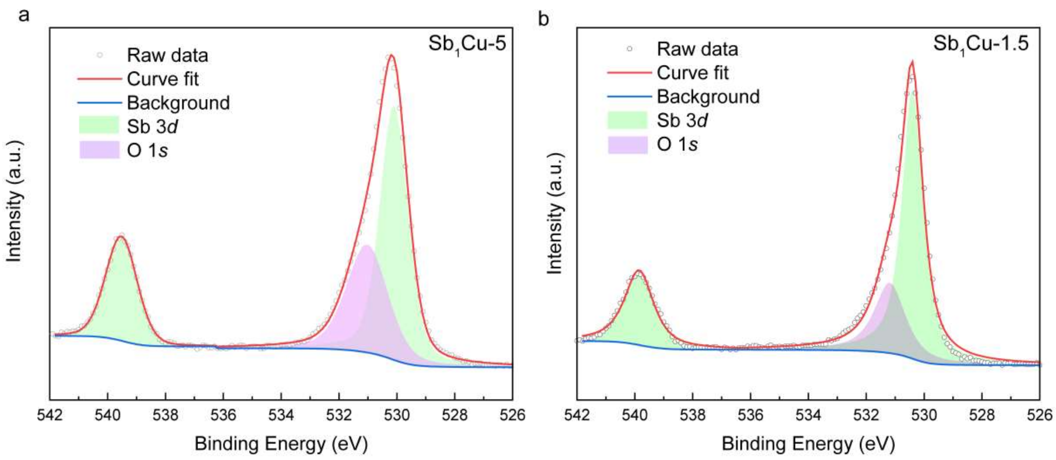

Supplementary Fig. 5 | Sb 3*d* XPS spectra of as-prepared a) Sb_1Cu-5 and b) Sb_1Cu-1.5 catalysts. The peaks at approximately 539.7 eV and 530.3 eV were assigned to Sb $3d_{3/2}$ and Sb $3d_{5/2}$, respectively, confirming the oxidation state of Sb^{III}.

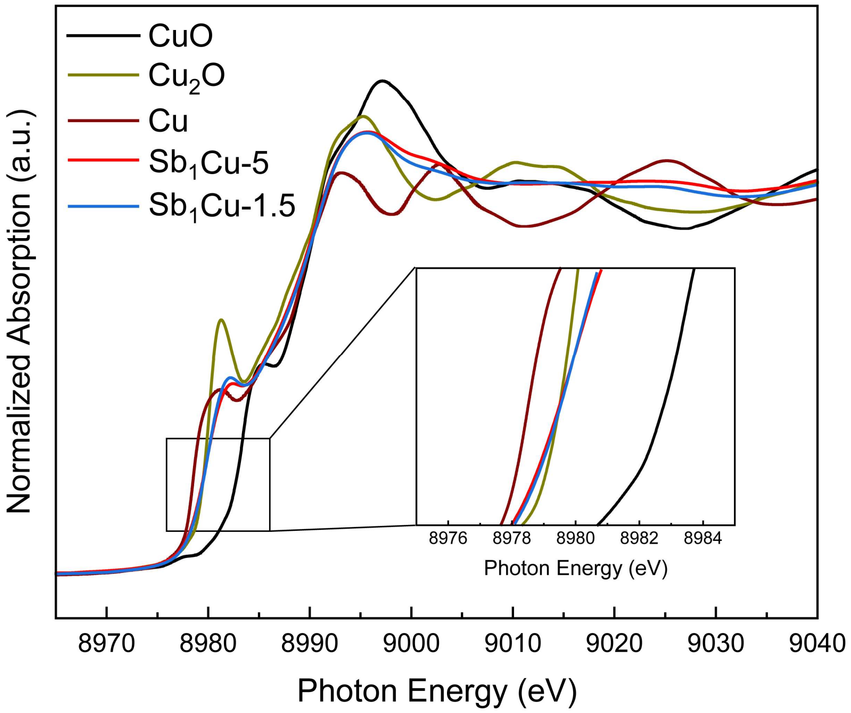

Supplementary Fig. 6 | Cu *K*-edge XANES spectra of as-prepared Sb_1Cu catalysts. Cu foil, Cu_2O and CuO were used as references for Cu^0, Cu^I and Cu^{II}, respectively. The near edge positions of the catalysts were consistent with the Cu_2O standard sample (inset of the figure), confirming the oxidation state of Cu^I in the as-synthesized Sb_1Cu catalysts.

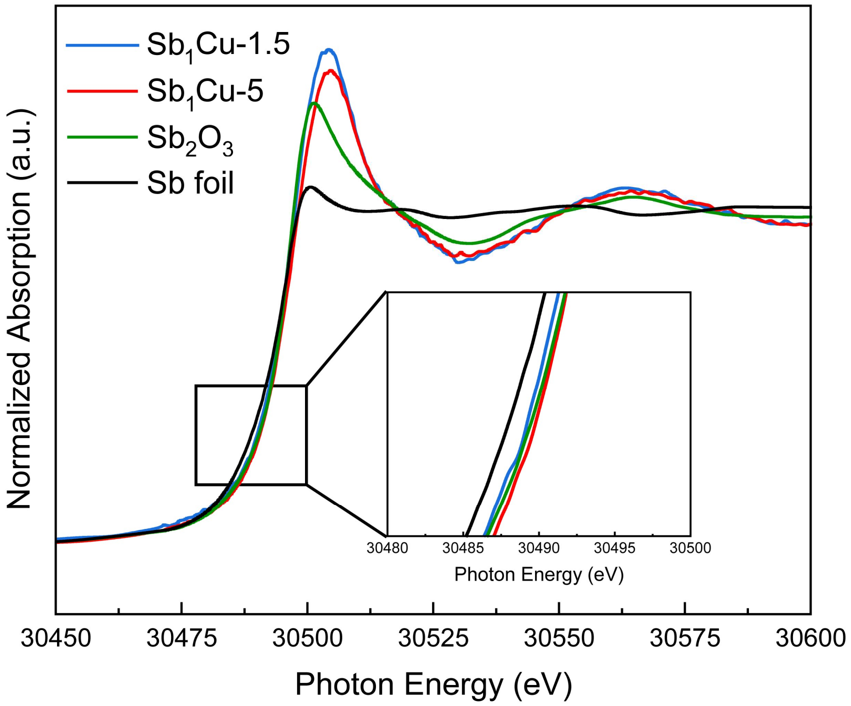

Supplementary Fig. 7 | Sb *K*-edge XANES spectra of as-prepared Sb_1Cu catalysts. Sb foil and Sb_2O_3 were used as references for Sb^0, Sb^{III}, respectively. The near edge positions of the catalysts were consistent with the Sb_2O_3 standard sample (inset of the figure), confirming the oxidation state of Sb^{III} in the as-synthesized Sb_1Cu samples.

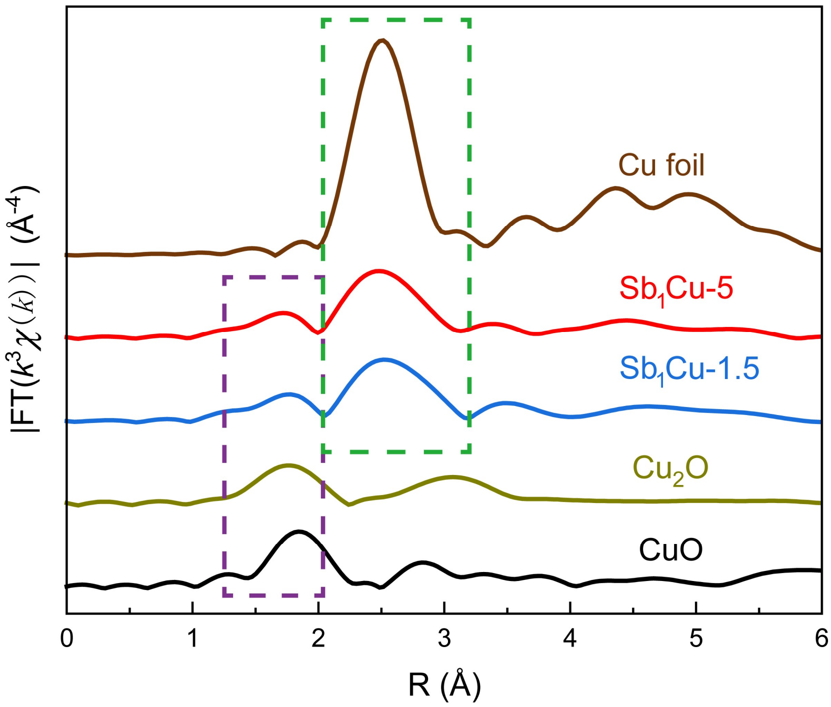

Supplementary Fig. 8 | EXAFS spectra at the Cu *K*-edge of the as-prepared Sb_1Cu catalysts. Cu, CuO and Cu_2O were used for references. The major peaks at approximately 2.50 Å were assigned to the Cu-Cu bond (highlighted in the green rectangle), while the Cu-O bond (peaks at approximately 1.75 Å, highlighted in the purple rectangle) was also observed due to partial oxidation in air, which was consistent with the XRD and XPS results.

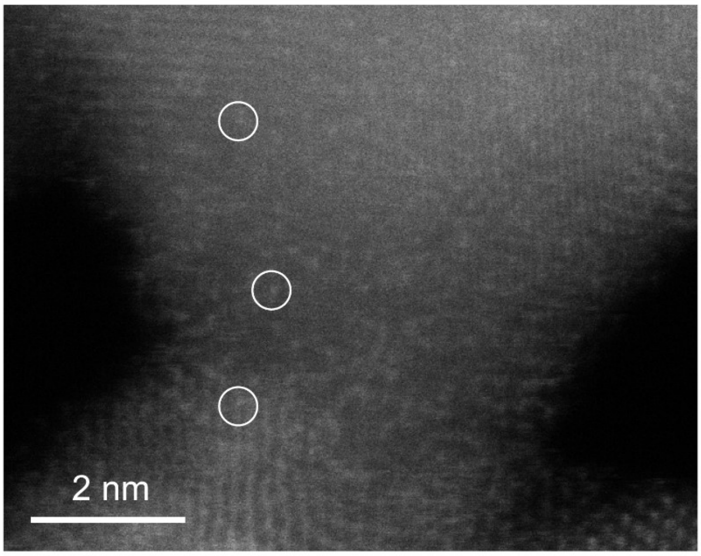

Supplementary Fig. 9 | HAADF-STEM image of the as-prepared Sb_1Cu-1.5 catalyst. White circles highlight isolated Sb atoms.

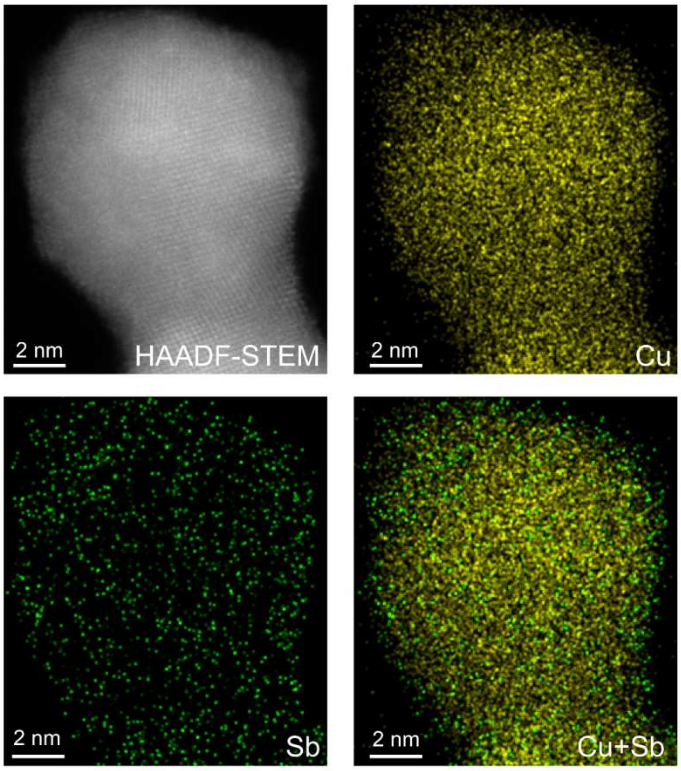

Supplementary Fig. 10 | STEM-EDS mapping of the as-prepared Sb_1Cu-1.5 catalyst. The image shows an even distribution of Sb in the Cu host.

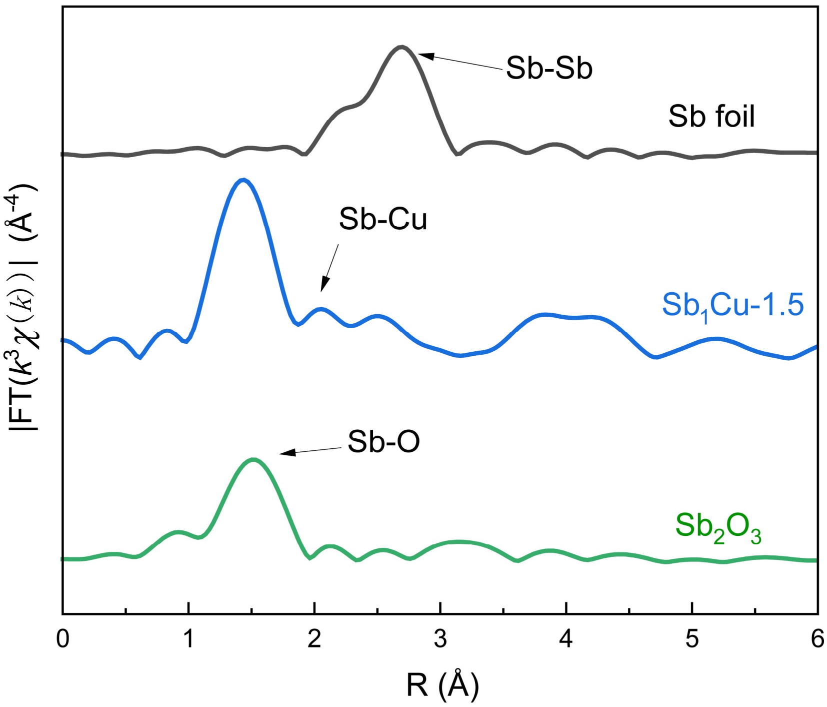

Supplementary Fig. 11 | Sb *K*-edge EXAFS spectra of the as-prepared Sb_1Cu-1.5 catalyst without phase correction. Sb foil and Sb_2O_3 are used as references. Peaks assigned to the Sb-Cu bond, together with the absence of the Sb-Sb bond, proved atomic Sb-Cu interfaces in the as-prepared Sb_1Cu-1.5 catalyst.

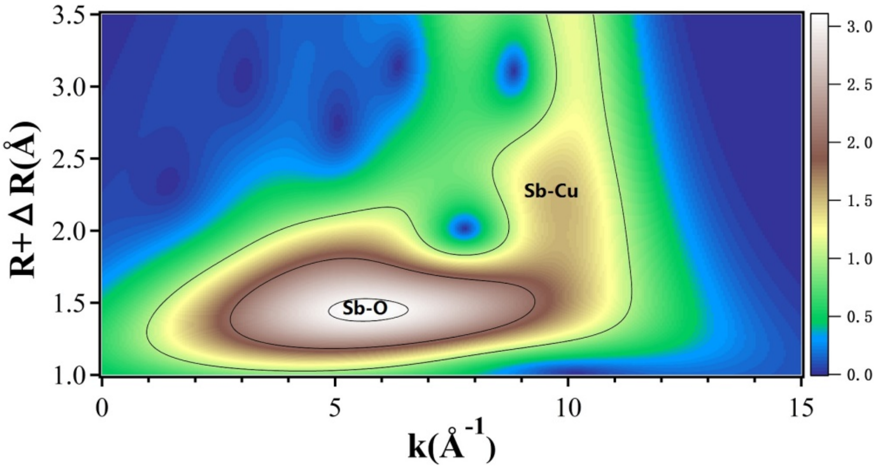

Supplementary Fig. 12 | EXAFS WT for the Sb_1Cu-1.5 catalyst. The Y-axis of the WT plots shows the radical distance, and the X-axis reflects the k-space resolution of the backscattering atom. Both the Sb-O bond and Sb-Cu bond can be clearly observed, verifying the formation of Sb_1Cu interfaces.

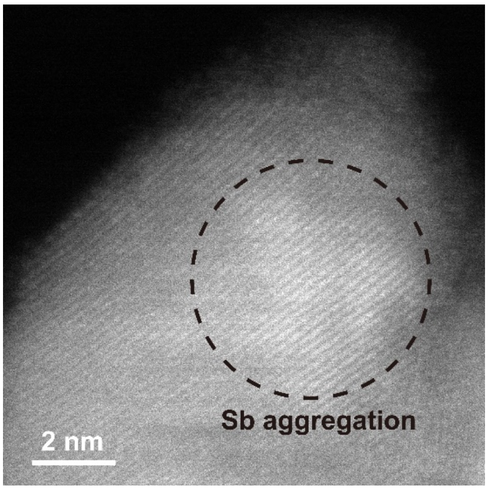

Supplementary Fig. 13 | HAADF-STEM image of the Sb_1Cu-10 catalyst. The black circle highlights Sb aggregation, confirming the formation of Sb clusters in the Sb_1Cu-10 catalyst.

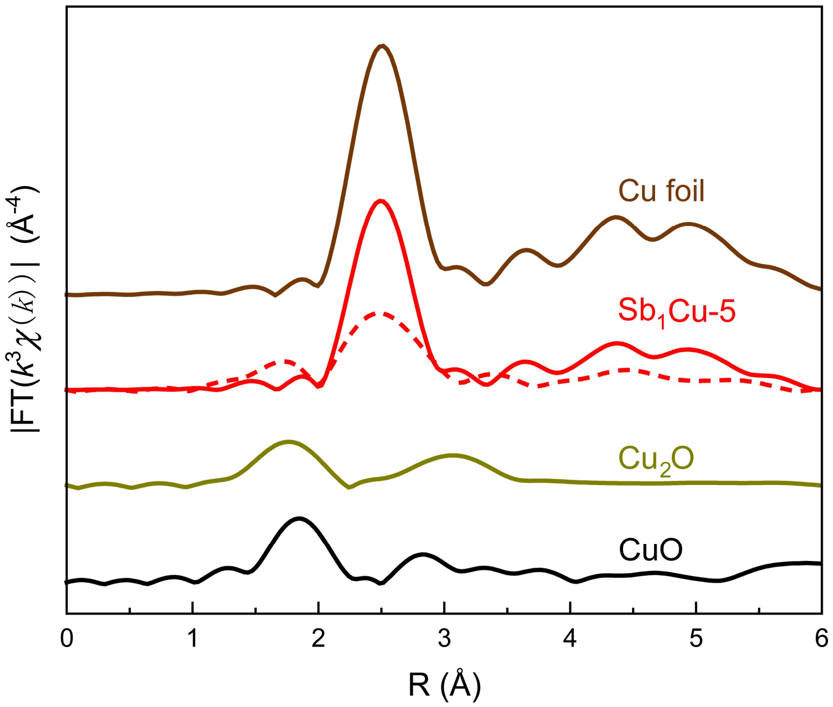

Supplementary Fig. 14 | *In situ* Cu *K*-edge EXAFS spectra of the Sb_1Cu-5 catalyst under CO_2RR conditions. Spectra of Sb_1Cu-5 under OCP (dashed line) and -1.0 V *vs.* RHE (solid line) are shown in the figure, with Cu, Cu_2O and CuO as references. The strengthened Cu-Cu peak (at approximately 2.50 Å) and disappearing Cu-O peak reconfirmed that metallic Cu was formed under the CO_2RR.

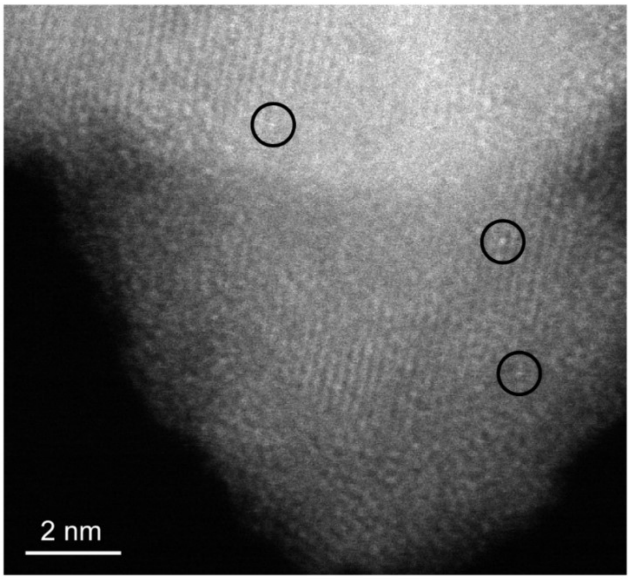

Supplementary Fig. 15 | Post-catalysis HAADF-STEM images of the Sb_1Cu-5 catalyst. The Sb single atoms were still mono-dispersed among the Cu matrix after CO_2RR, highlighted in black circles.

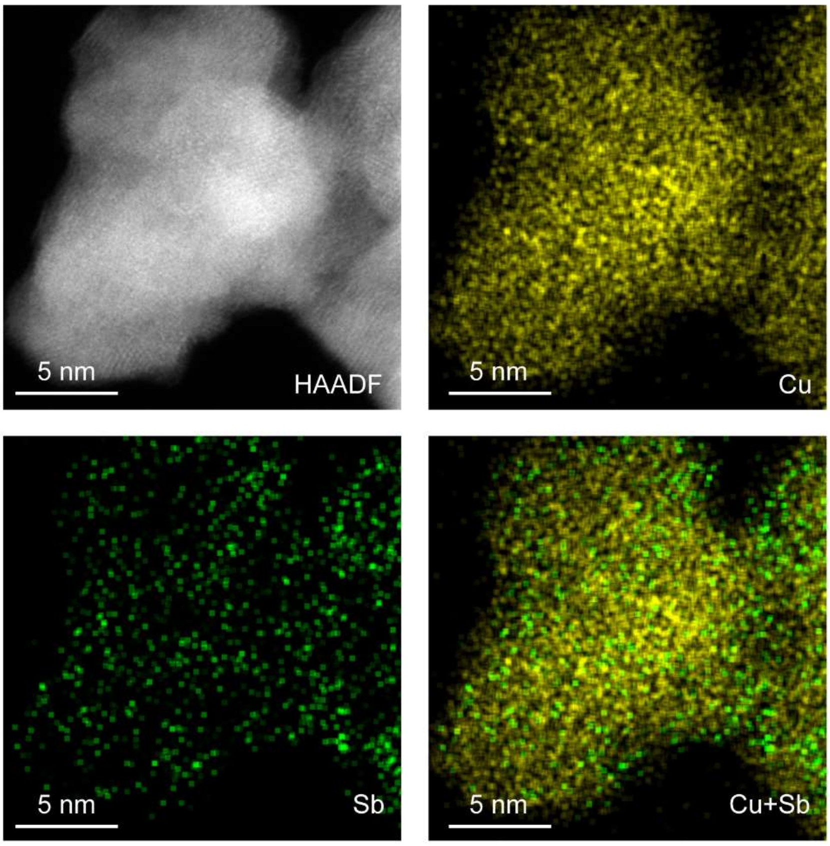

Supplementary Fig. 16 | STEM-EDS mapping of the Sb_1Cu-5 catalyst after long-term electrolysis. The homogeneous distribution of Sb in the Cu matrix was maintained after the CO_2RR.

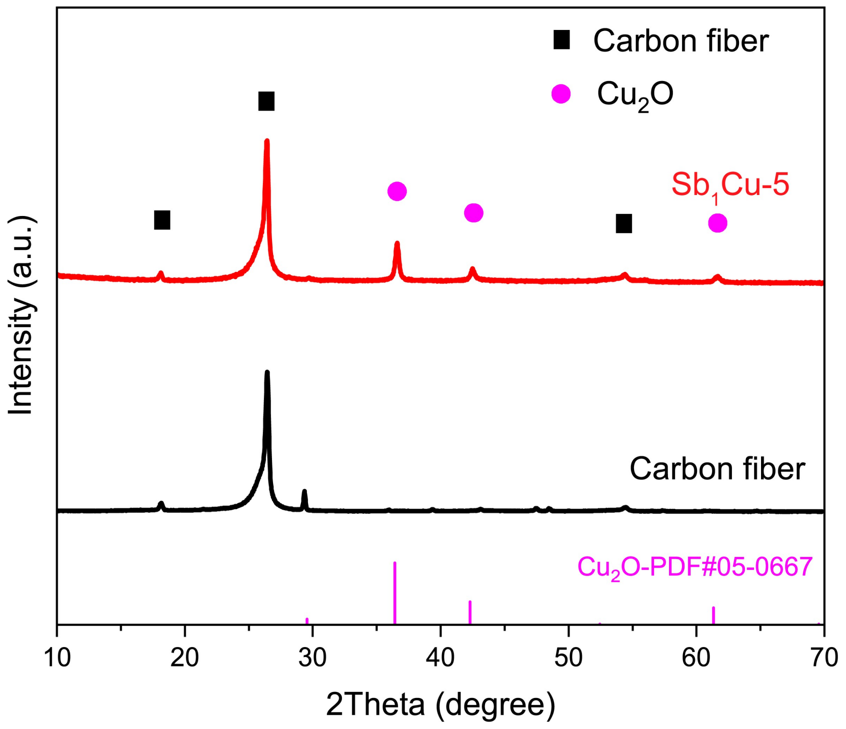

Supplementary Fig. 17 | XRD pattern of post-catalysis Sb_1Cu-5 catalyst on the GDL. The peaks of carbon fiber from the GDL were excluded, and the spectra were consistent with the Cu_2O reference because of the rapid oxidization in air during *ex situ* XRD measurements.

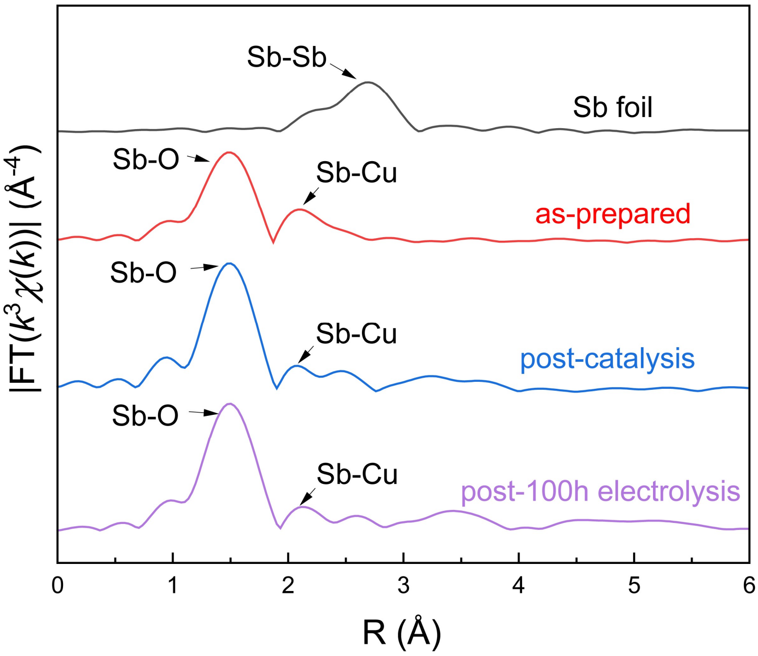

Supplementary Fig. 18 | *Ex situ* Sb *K*-edge EXAFS spectra of the Sb_1Cu-5 catalyst after 100 h of continuous electrolysis without phase correction. Sb foil is used as a reference. Peaks assigned to the Sb-Cu bond, together with the absence of the Sb-Sb bond, proved robust atomic Sb-Cu interfaces in the Sb_1Cu-5 catalyst after 100 hours of continuous electrolysis. In addition, the as-prepared sample and the sample after 30 min of electrolysis in the flow cell are also shown for comparison. The Sb_1Cu-5 catalyst after 100 hours of electrolysis in the H-cell showed almost the same Sb coordination structure as the as-prepared and post-catalysis samples, demonstrating the intrinsic long-term stability of the catalyst.

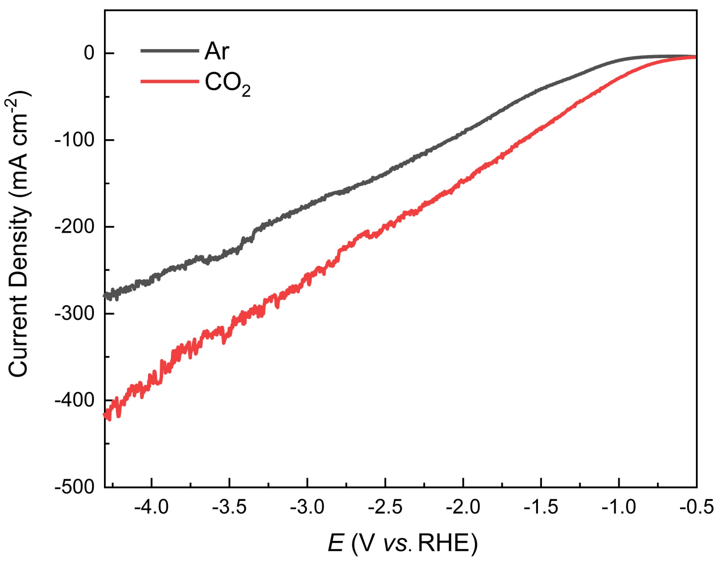

Supplementary Fig. 19 | LSV of Sb_1Cu-5 catalyst. The polarized curves were collected in a conventional three-electrode flow cell system using a scan rate of 0.1 V s^{-1}. 0.7 mL min^{-1} 0.5 M $KHCO_3$ electrolyte and 30 sccm CO_2 or Ar flow were provided. The values of potential were presented without i-R compensation.

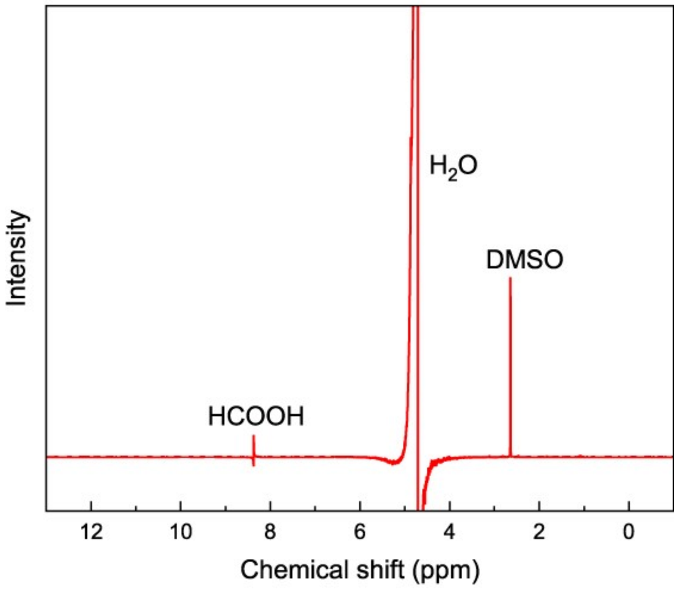

Supplementary Fig. 20 | Typical ^{1}H NMR spectrum of liquid product for CO_2RR on Sb_1Cu-5 catalyst. No other peak except HCOOH and DMSO (internal standard) was observed, proving that HCOOH was the only liquid product on the Sb_1Cu-5 catalyst.

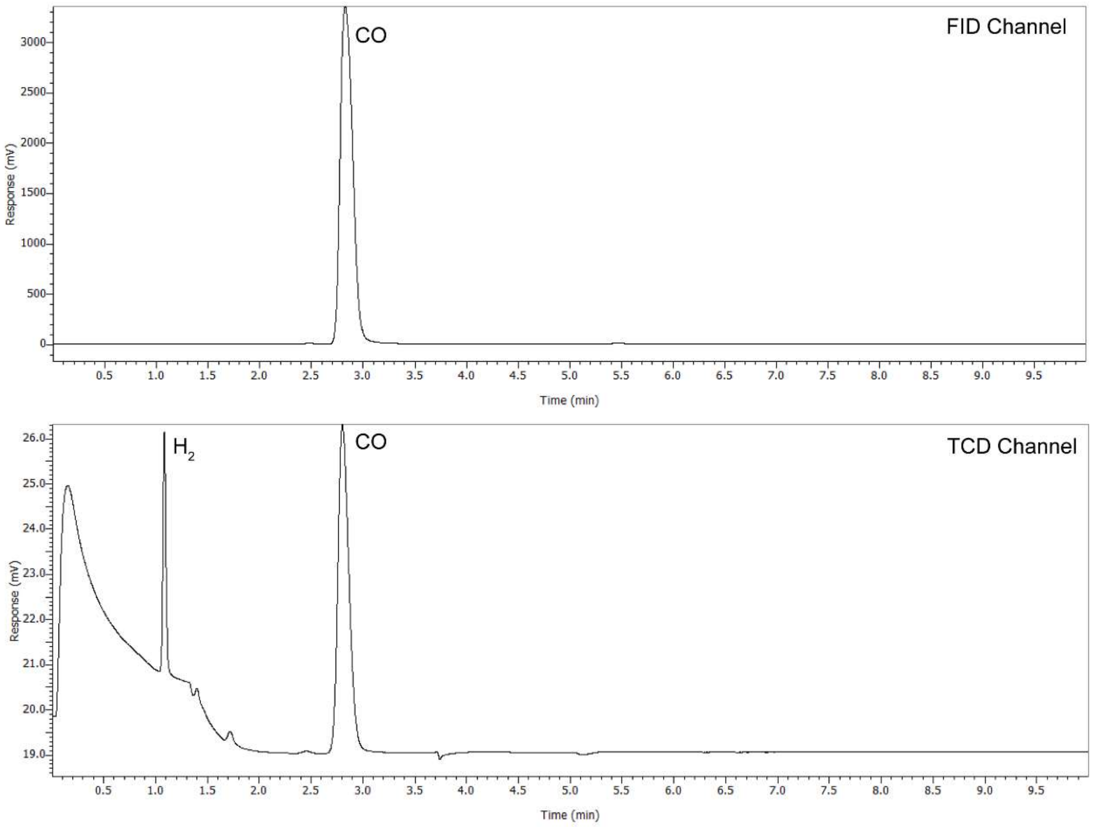

Supplementary Fig. 21 | Typical GC response to gas products of the CO_2RR on the Sb_1Cu-5 catalyst. The GC spectra verified H_2 and CO as the gas products, excluding CH_4 and C_2H_4 production.

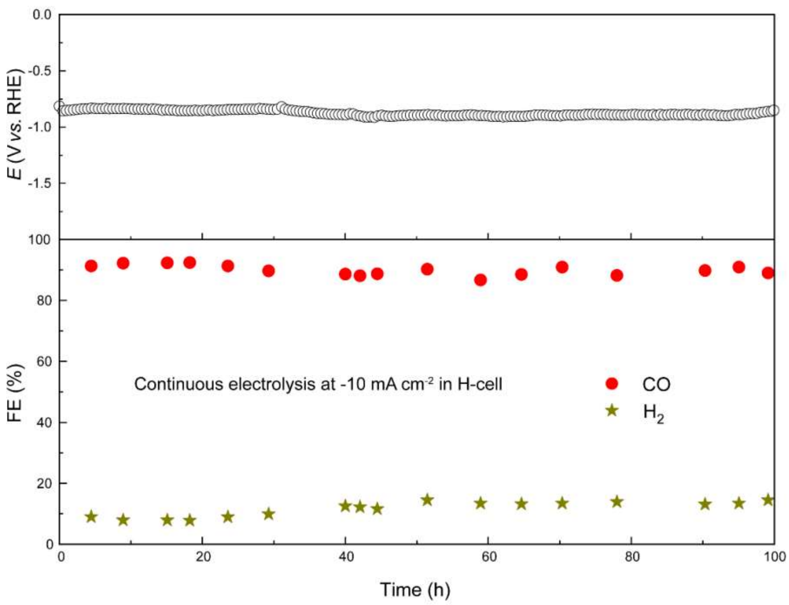

Supplementary Fig. 22 | Stability test at a current density of -10 mA cm^{-2} in an H-cell for 100 h. To explore the intrinsic stability of Sb_1Cu-5 catalysts, we used an H-cell as the reaction reactor, which could avoid flooding and carbonation problems in MEA. The FE_{CO} of approximately 90% during 100 h of continuous electrolysis and the stable cathode potential confirmed the impressive stability of the catalyst.

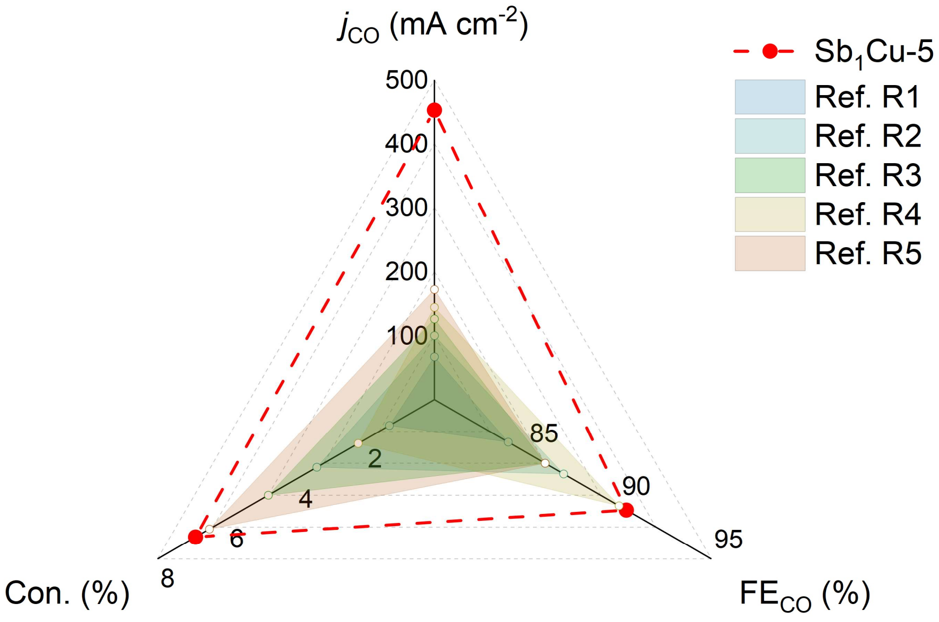

Supplementary Fig. 23 | Comparison of the CO partial current density (j_{CO}), CO Faradaic efficiency (FE_{CO}) and conversion rate of CO_2-to-CO (Con.) with those of state-of-the-art CO-selective electrocatalysts. [Ref. R1: *Angew. Chem. Int. Ed.* **61**, e202111683 (2022)]; [Ref. R2: *Nat. Commun.* **12**, 1449 (2021)]; [Ref. R3: *Angew. Chem. Int. Ed.* **60**, 11959-11965 (2021)]; [Ref. R4: *ACS Energy Lett.* **3**, 2835-2840 (2018)]; [Ref. R5: *Science* **365**, 367–369 (2019)]

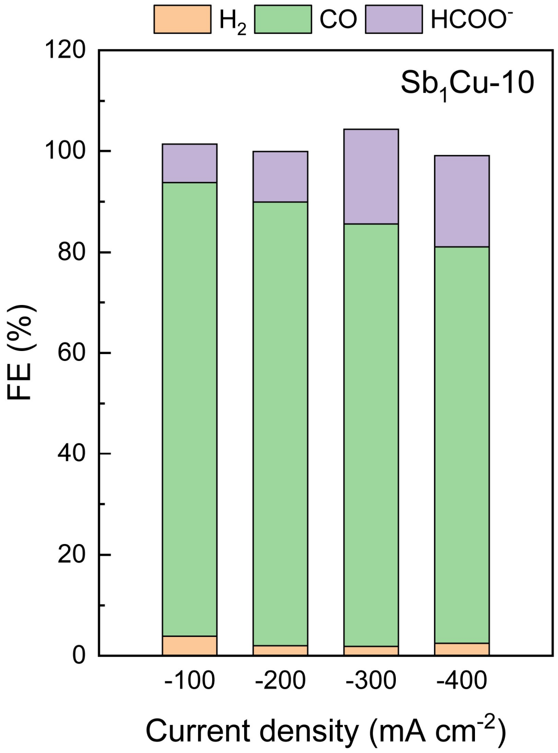

Supplementary Fig. 24 | CO_2RR performance of the Sb_1Cu-10 catalyst in a flow cell. As expected, due to the formation of Sb-Cu interfaces, negligible C_{2+} formation and over 80% FE_{CO} were found on Sb_1Cu-10, which confirmed the role of Sb-Cu interfaces in facilitating CO desorption and limiting C-C coupling. However, more formate produced on Sb_1Cu-10 compared with Sb_1Cu-5 was attributed to the formation of Sb clusters, considering that pure Sb exhibited relatively higher selectivity towards formate. This result manifested the importance of isolated Sb-Cu interfaces.

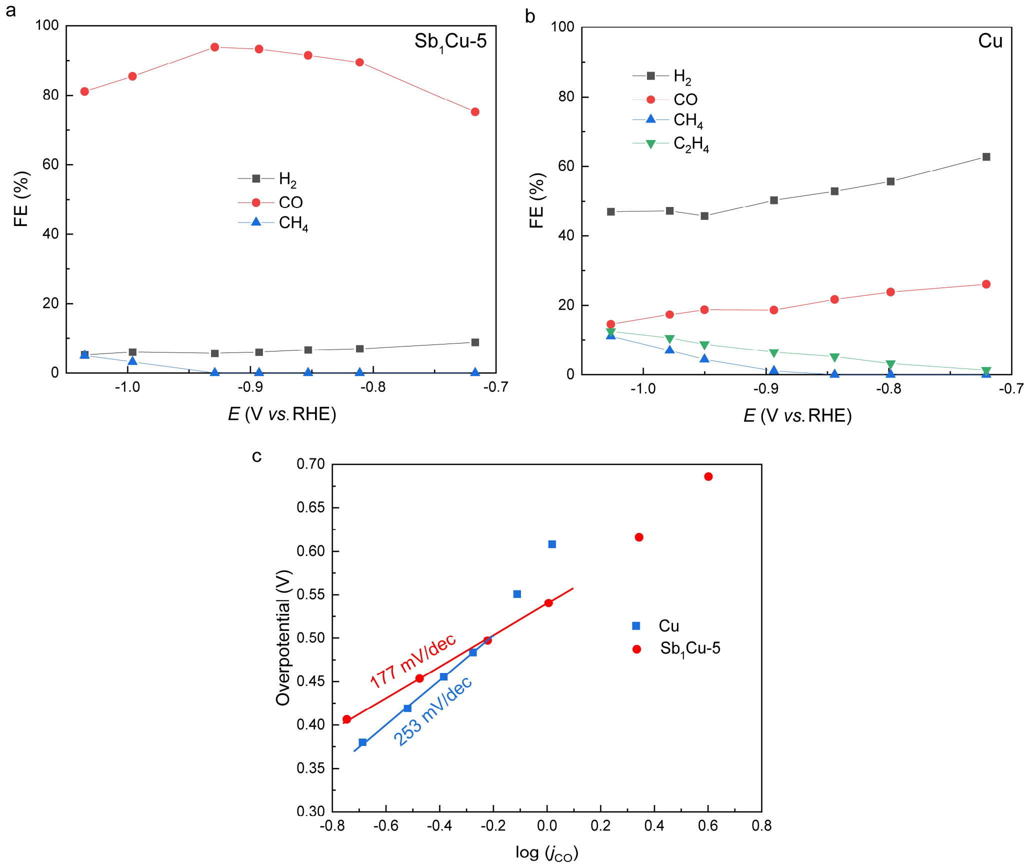

Supplementary Fig. 25 | a, b) H-cell performance and c) Tafel plot of Sb_1Cu-5 and Cu catalysts. In the H-cell, FE_{CO} on Sb_1Cu-5 reached a maximum of 93.8% at -0.93 V and maintained >80% over a wide potential window (-0.8 ~ -1.15 V). In contrast, FE_{CO} remained below 30% on Cu, and C_2H_4 production was observed, indicating C-C coupling. In the Tafel plot, the smaller slope of the Sb_1Cu-5 catalyst indicated faster kinetics towards the CO_2RR to CO. Supplementary Table 5 shows the standard values of the Tafel slopes. A Tafel slope larger than 118 mV dec^{-1} indicated the RDS of the first electron transfer CO_2-to-$*CO_2^-$. Deviation from the standard value was attributed to the asymmetry factor ($\alpha \neq 0.5$).

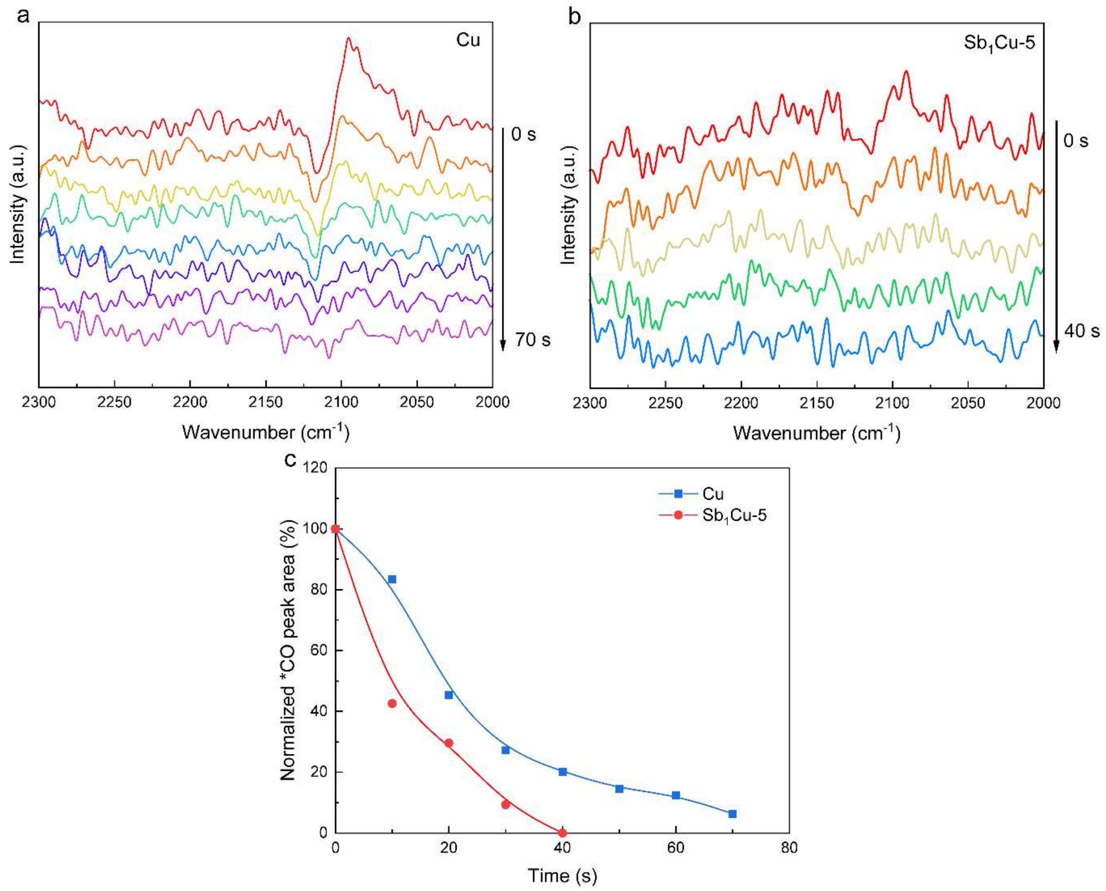

Supplementary Fig. 26 | *In situ* ATR-SEIRAS spectra of a) Cu and b) Sb_1Cu-5 under an Ar sweep after suspension of the applied potential and c) attenuation of the *CO peak area with time. To further confirm the better ability of CO desorption on Sb_1Cu-5, we investigated the *CO retention time under an Ar sweep. The faster attenuation rate and shorter retention time of *CO on Sb_1Cu-5 than Cu manifested its lower binding energy of *CO and better ability of CO desorption.

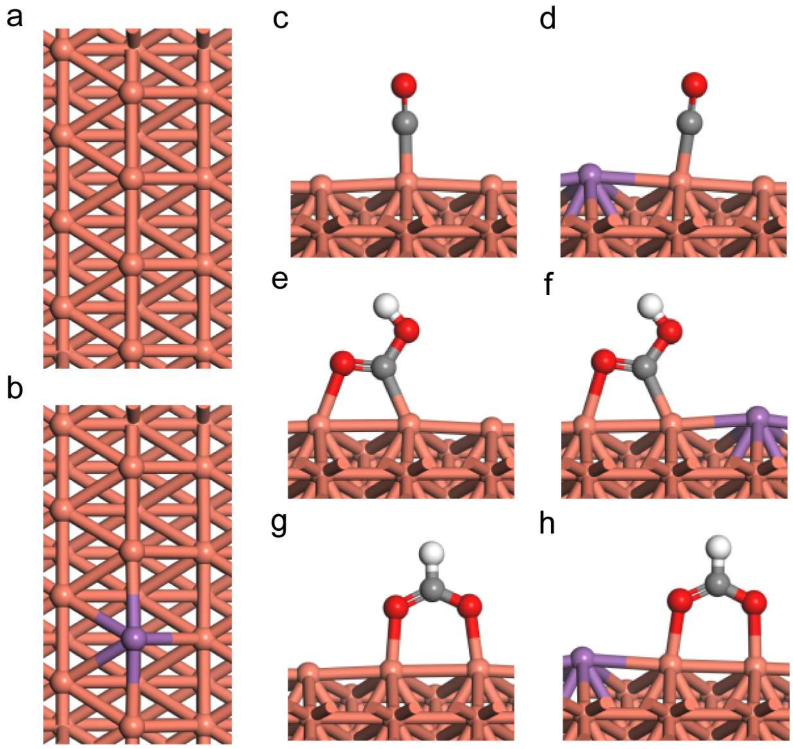

Supplementary Fig. 27 | Surface structures. Cu, Sb, C, O and H are represented in orange, purple, gray, red and white, respectively. The surface structures of a) Cu (211) and b) Sb_1Cu-5 (211). The adsorption states of CO* on c) Cu (211) and d) Sb_1Cu-5 (211). The adsorption states of COOH* on e) Cu (211) and f) Sb_1Cu-5 (211). The adsorption states of HCOO* on g) Cu (211) and h) Sb_1Cu-5 (211).

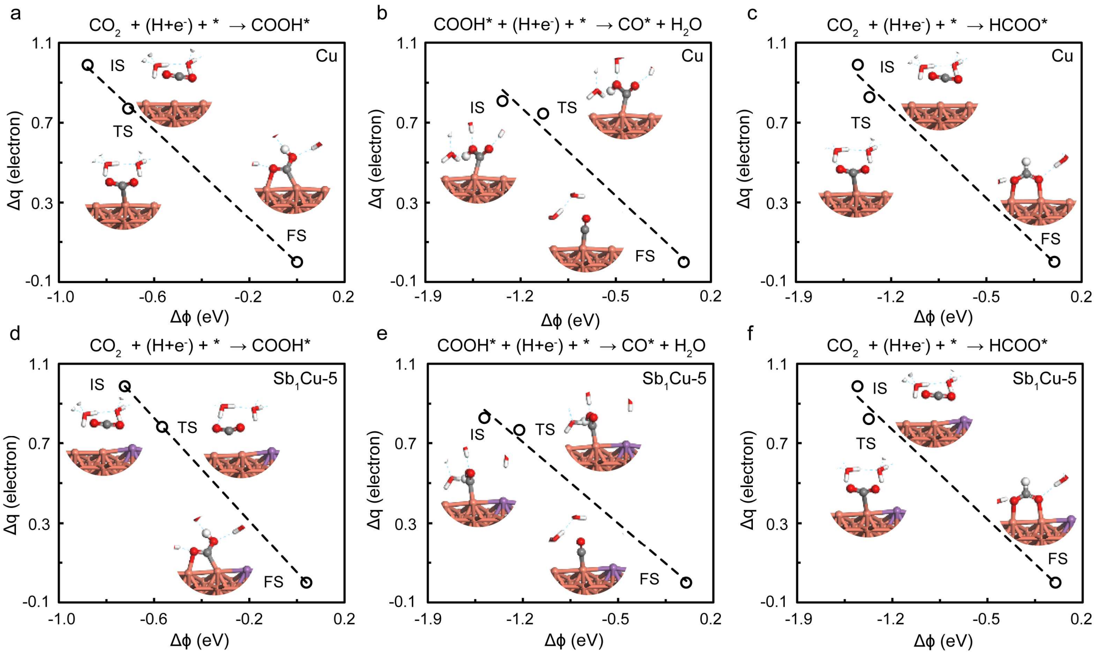

Supplementary Fig. 28 | The scheme of 'charge-extrapolation'. Linear relations between the amount of electrons transferred from the electrode to the water layer (Δq) and the relative work function of the system ($\Delta \Phi$) at the initial (IS), transition (TS) and final (FS) states for CO_2 protonation and CO* formation. Cu, Sb, C, O and H are represented in orange, purple, gray, red and white, respectively.

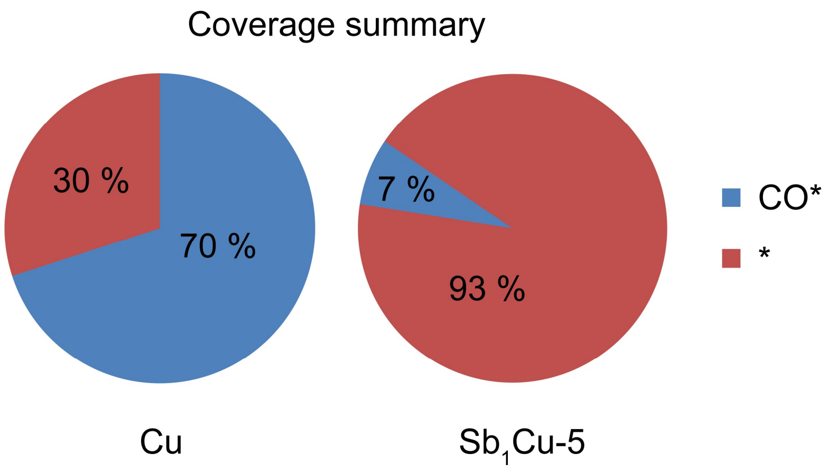

Supplementary Fig. 29 | Coverage summary at the steady state for the CO_2RR on Cu (211) and Sb_1Cu-5 (211). The CO* coverage takes up 70% of the total sites for the CO_2RR on pristine Cu, providing a much higher possibility for C-C coupling. In contrast, CO* occupies only 7% coverage on Sb_1Cu-5 (211). The results are consistent with *in situ* ATR-SEIRAS and *in situ* Raman measurements.

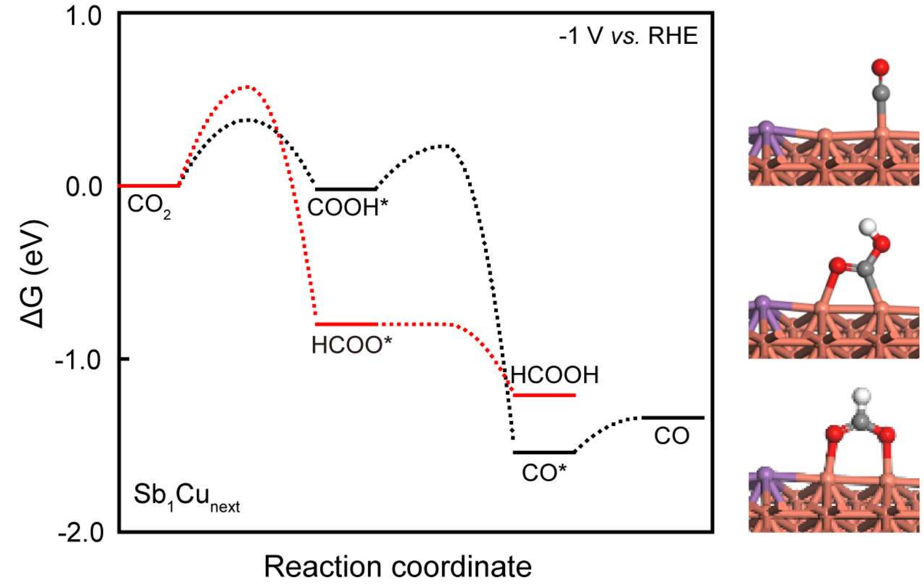

Supplementary Fig. 30 | CO_2RR to CO and HCOOH on Cu site (next-nearest to Sb atom) on Sb_1Cu-5 (211). The adsorption structures are shown on the right, where Cu, Sb, C, O and H are represented in orange, purple, gray, red and white, respectively.

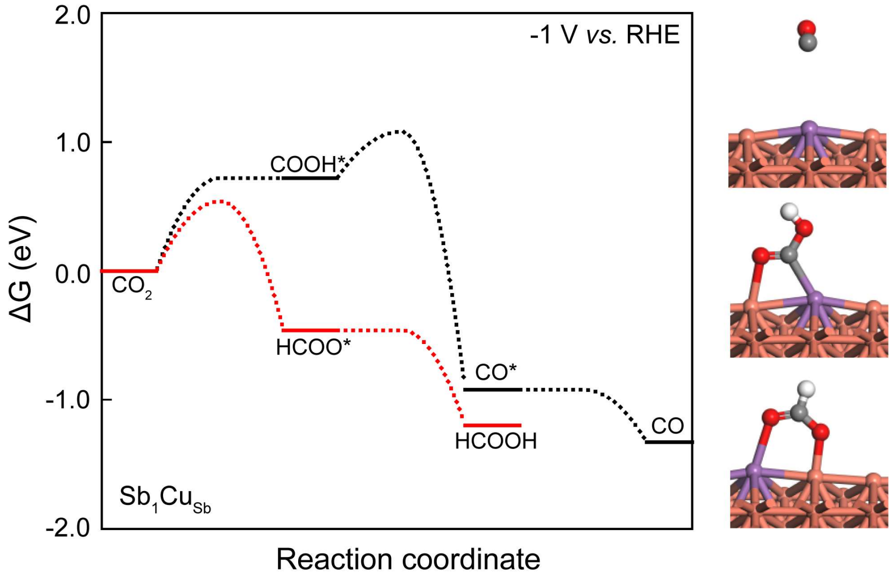

Supplementary Fig. 31 | CO_2RR to CO and HCOOH on Sb_1Cu-5(211)$_{Sb}$. The adsorption structures are shown on the right, where Cu, Sb, C, O and H are represented in orange, purple, gray, red and white, respectively. This revealed that the different concentrations of Sb may be the reason for the different products in different papers.

Supplementary Table 1 | EXAFS fitting parameters at the Sb *K*-edge for Sb_1Cu samples before and after the CO_2RR (S_0^2=0.938).

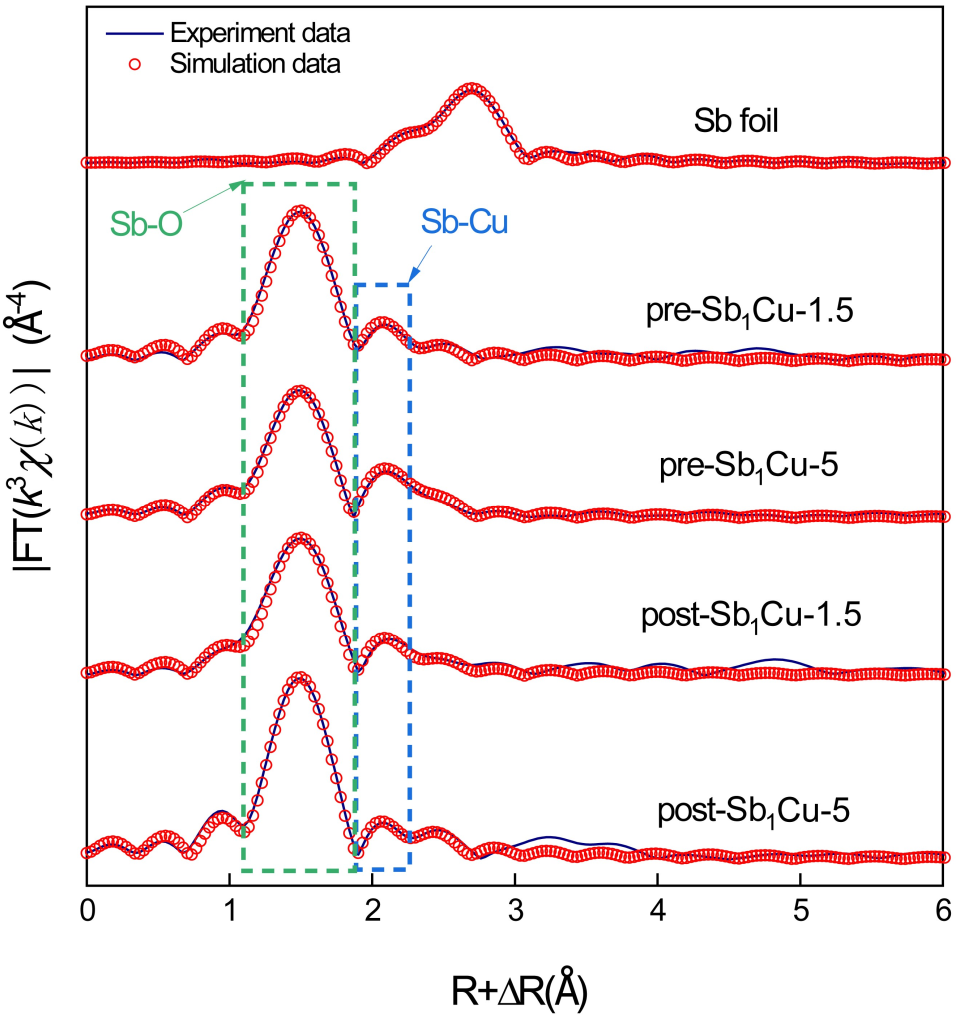

Sample	Shell	N^a	R(Å)^b	σ^2(Å^2)^c	ΔE_0(eV)^d	R factor
Sb foil	Sb-Sb	3	2.91	0.0039	8.3	0.0045
pre-Sb_1Cu-1.5	Sb-O	5.4	1.98	0.0022	8.0	0.0015
	Sb-Cu	0.7	2.68	0.0071		
pre-Sb_1Cu-5	Sb-O	4.8	1.98	0.0029	7.9	0.0015
	Sb-Cu	2.4	2.64	0.0102		
post-Sb_1Cu-1.5	Sb-O	5.8	1.99	0.0037	8.3	0.0039
	Sb-Cu	0.9	2.68	0.0068		
post-Sb_1Cu-5	Sb-O	5.9	1.98	0.0018	9.9	0.0007
	Sb-Cu	1.0	2.58	0.0087		

$^a N$: coordination numbers; $^b R$: bond distance; $^c \sigma^2$: Debye-Waller factors; $^d \Delta E_0$: inner potential correction. R factor: goodness of fit. S_0^2 was set to 0.938 for Sb, according to the experimental EXAFS fit of the Sb foil reference by fixing CN as the known crystallographic value.

Supplementary Table 2 | Performance of recently reported non-Cu-based CO_2-to-CO electrocatalysts in flow cells.

Catalyst	Potential (V *vs.* RHE)	j_{CO} (mA cm^{-2})	FE_{CO} (%)	Ref.
**Sb_1Cu-5**	**-1.16**	**452**	**90.4**	**This work**
	**-1.13**	**360**	**90.0**	
Ni-SA/PCFM	-1.2	337	81	*Nat. Commun.* **11**, 593 (2020)
Fe^{3+}-N-C	-0.45	94	94	*Science* **164**, 1091-1094 (2019)
CoPc@Fe-N-C	-0.83	277	94	*Adv. Mater.* **31**, 1903470 (2019)
Ni@NiNCM	-0.92	126	84	*Angew. Chem. Int. Ed.* **60**, 11959-11965 (2021)
CoPc	/	172	86	*Science* **365**, 347-369 (2019)
Mg-C_3N_4	-0.61	270	90	*Angew. Chem. Int. Ed.* **60**, 25241-25245 (2021)
Zn/NC-NSs	-1.06	67	84	*Angew. Chem. Int. Ed.* **61**, e202111683 (2022)

Supplementary Table 3 | Performance of recently reported Cu-based CO_2-to-CO electrocatalysts.

Catalyst	Potential (V *vs.* RHE)	j_{CO} (mA cm^{-2})	FE_{CO} (%)	Ref.
**Sb_1Cu-5**	**-1.16**	**452**	**90.4**	**This work**
	**-1.13**	**360**	**90.0**	
Cu/$Ni(OH)_2$	-0.5	3.7	92	*Sci. Adv.* **3**, 9 (2017)
Cu-APC	-0.78	8.6	92	*Nat. Chem.* **11**, 222-228 (2019)
Cu-S_1N_3/Cu_x	-0.75	7.5	90	*Angew. Chem. Int. Ed.* **60**, 24022–24027 (2021)
V-$CuInSe_2$	-0.6	70	92	*Adv. Mater.* **34**, 2106354 (2022)
$CuCo_{1.0}$	/	60.5	97.4	*ACS Sustain. Chem. Eng.* **8**, 12561−12567 (2020)
CuZn NW	-1.0	14.4	90	*ACS Catal.* **12**, 2741−2748 (2022)
$Cu_{97}Sn_3$	-0.45	120	87	*Nat. Commun.* **12**, 1449 (2021)
Cu/Cu_2O-Sb-5	/	110	91	*J. Mater. Chem. A* **9**, 23234 (2021)

Supplementary Table 4 | Performance of recently reported Sb-Cu electrocatalysts in an H-cell.

Catalyst	Potential (V *vs.* RHE)	j_{CO} (mA cm^{-2})	Mass activity (mA mg^{-1})	FE_{CO} (%)	Stability (h)	Ref.
Sb_1Cu-5	**-0.85**	**9.2**	**9.2**	**92.3**	**100**	**This work**
Cu_2Sb NA/CF	-0.9	6	-	86.5	2	*Nano Res.* **14**, 2831-2836 (2021)
$Sb_{0.22}Cu$	-0.8	16.2	5.4	95	10	*Appl. Catal. B: Environ.* **306**, 121089 (2022)
Sb-Cu	-1.1	4.7	2.9	80	12	*ACS Catal.* **11**, 6846-6856 (2021)

Supplementary Table 5 | Tafel slope for different possible RDSs during the CO_2RR to CO.

**Possible RDS**	**Type[a]**	**Tafel slope (mV dec^{-1})[b]**
$CO_2 + * + e^- \rightarrow *CO_2^-$	ET	118
$*CO_2^- + H_2O \rightarrow *COOH + OH^-$	PT	59
$*COOH + e^- \rightarrow *COOH^-$	ET	39
$*COOH^- \rightarrow *CO + OH^-$	PT	30
$*CO \rightarrow CO + *$	D	30

[a]*ET* electron transfer, *PT* proton transfer, *D* desorption

[b]Assuming $\alpha = 0.5$

Supplementary Table 6 | Elementary steps for FTacV simulations and key fitting parameters.

Type	Elementary steps	Fitting parameters
Electron transfer	$CO_2 + * + e^- \rightarrow *CO_2^-$	$k_s = 6.5$ cm s^{-1}<br>$\alpha = 0.34$
Chemical reaction	$*CO_2^- + H_2O \rightarrow *COOH + OH^-$	$k_f = 2.5\times10^3$ $L\cdot mol^{-1}\cdot s^{-1}$<br>$k_b = 1.5$ $L\cdot mol^{-1}\cdot s^{-1}$
Electron transfer	$*COOH + e^- \rightarrow *COOH^-$	$k_s = 7$ cm s^{-1}<br>$\alpha = 0.37$
Chemical reaction	$*COOH^- \rightarrow *CO + OH^-$	$k_f = 2.0\times10^4$ s^{-1}<br>$k_b = 1.5$ $L\cdot mol^{-1}\cdot s^{-1}$
k_s standard rate constant, α transfer coefficient for electron transfer steps;<br>k_f forward rate constant, k_b backward rate constant for chemical reactions.		
